# New Mutations Involved in Colistin Resistance in Acinetobacter baumannii

**DOI:** 10.1128/mSphere.00895-19

**Published:** 2020-04-01

**Authors:** Bingbing Sun, Haiyan Liu, Yu Jiang, Lei Shao, Sheng Yang, Daijie Chen

**Affiliations:** a School of Pharmacy, Shanghai Jiao Tong University, Shanghai, China; b State Key Laboratory of Microbial Metabolism, Shanghai Jiao Tong University, Shanghai, China; c Key Laboratory of Synthetic Biology, CAS Center for Excellence in Molecular Plant Sciences, Shanghai Institute of Plant Physiology and Ecology, Chinese Academy of Sciences, Shanghai, China; d State Key Laboratory of Anti-Infective Drug Development, Sunshine Lake Pharma Company, Ltd., Dongguan, China; e College of Engineering, China Pharmaceutical University, Nanjing, China; f Shanghai University of Medicine and Health Sciences, Shanghai, China; g Huzhou Center of Industrial Biotechnology, Shanghai Institutes of Biological Sciences, Chinese Academy of Sciences, Shanghai, China; JMI Laboratories

**Keywords:** *Acinetobacter baumannii*, colistin, mechanism of resistance

## Abstract

Colistin is used as the “last resort” to treat infections caused by multidrug-resistant Acinetobacter baumannii, which is at the top of the World Health Organization’s list of the most dangerous bacterial species that threaten human health. Unfortunately, colistin resistance has emerged in A. baumannii. To broaden the study of the resistance mechanism of colistin in A. baumannii, we obtained colistin-resistant mutants via two methods: (i) screening and isolation from a *mariner*-based A. baumannii ATCC 19606 transposon mutant library; (ii) selection from challenge of ATCC 19606 with successively increasing concentrations of colistin. A total of 41 mutants with colistin MIC of 4 μg/ml to 64 μg/ml were obtained by transposon mutant library screening. Five highly resistant mutants with colistin MICs ranging from 256 μg/ml to 512 μg/ml were selected from successive colistin challenges. Genotypic complementation and remodeling of the transposon mutants revealed that the genes inactivated by the transposon insertion were not responsible for resistance. Whole-genome sequence analysis of the colistin-resistant strains revealed that the main causes of the resistance to colistin were mutations in the *pmrA-pmrB* genes, including *pmrA*^P102R^, *pmrB*^P233S^, and *pmrB*^T235N^ and the novel alleles *pmrA*^I13M^ and *pmrB*^Q270P^. Interestingly, we found that *miaA*^I221V^ mutation of A. baumannii strain ATCC 19606 (*pmrA*^P102R^) resulted in 4-fold increases in the colistin MIC, which rose from 32 μg/ml to 128 μg/ml. But *miaA*^I221V^ itself had little effect on the colistin susceptibility of ATCC 19606. These data broaden knowledge of the scope of chromosomally encoded mechanisms of resistance to colistin.

**IMPORTANCE** Acinetobacter baumannii is an important Gram-negative opportunistic pathogen commonly infecting critically ill patients. It possesses a remarkable ability to survive in the hospital environment and acquires resistance determinants corresponding to a wide range of antibacterial agents. Given that the current treatment options for multidrug resistant A. baumannii are extremely limited, colistin administration has become the treatment of last resort. However, colistin-resistant A. baumannii strains have recently been reported. The mechanism of resistance to colistin in A. baumannii has rarely been reported. Here, we found two novel mutations in *pmrA* (I13M) and *pmrB* (Q270P) that caused colistin resistance. It is also first reported here that the presence of *miaA* with a I221V mutation enhanced the colistin resistance of *pmrA*^P102R^.

## Abstract (precis)

Acinetobacter baumannii is an important Gram-negative opportunistic pathogen commonly infecting critically ill patients. It possesses a remarkable ability to survive in the hospital environment and acquires resistance determinants corresponding to a wide range of antibacterial agents. Given that the current treatment options for multidrug resistant A. baumannii are extremely limited, colistin administration has become the treatment of last resort. However, colistin-resistant A. baumannii strains have recently been reported. The mechanism of resistance to colistin in A. baumannii has rarely been reported. Here, we found two novel mutations in *pmrA* (I13M) and *pmrB* (Q270P) that caused colistin resistance. It is also first reported here that the presence of *miaA* with a I221V mutation enhanced the colistin resistance of *pmrA*^P102R^.

## INTRODUCTION

Acinetobacter baumannii is a significant nosocomial pathogen that is also readily found in soil, water, and animals (1–3). A. baumannii has been implicated in a variety of nosocomial infections, including respiratory tract infections, urinary tract infections, secondary meningitis, genitourinary infections, and others (4). Until recently, A. baumannii showed susceptibility to most commonly used antibacterial drugs. However, due to the abuse of broad-spectrum antibacterial drugs, its resistance to antimicrobials, including sulfonamides, β-lactams, and aminoglycosides (5, 6), has been significantly enhanced since the late 1970s. Since the first report, in 1991, of carbapenem-resistant A. baumannii in the United States, this bacterium has become resistant to most antibiotics used in the clinical setting (7). In 2017, WHO has included carbapenem-resistant A. baumannii in its priority list of the top 12 pathogens (8).

As an “old” antibiotic, colistin was originally used to treat clinical bacterial infections. But it was replaced by other drugs due to severe nephrotoxicity and neurotoxicity in the 1970s (9). Recently, colistin has reemerged as a “choice-of-no-choice” for the treatment of infections by multidrug-resistant Gram-negative bacilli, especially the highly resistant species Pseudomonas aeruginosa and A. baumannii, in the Asia-Pacific region. The reemergence of the “old drug” is considered to represent the last resort for the treatment of infections by multidrug-resistant Gram-negative bacteria (10).

The mechanism of colistin antibacterial activity is still unclear. The currently recognized mechanism is that colistin binds to lipopolysaccharide (LPS) on the outer membrane of Gram-negative bacteria, causing the outer membrane to swell, which subsequently disrupts the phospholipid bilayer via a self-promoted uptake mechanism, leading to an osmotic imbalance that leads to cell death (11, 12).

Along with the clinical use of colistin for treatment of multidrug-resistant A. baumannii infections, colistin-resistant A. baumannii has emerged. Since colistin-resistant Acinetobacter spp. were first reported in the Czech Republic, in 1999 (13, 14), the incidence of resistance reported all over the world has been increasing year by year. The mechanism of A. baumannii resistance to colistin is mainly that of the modification of lipid A or the loss of lipopolysaccharide. In 2009, Adams et al. (15) compared the DNA sequences of the PmrA-PmrB two-component system (TCS) in A. baumannii colistin-sensitive and colistin-resistant strains and found mutations in *pmrA* and *pmrB* (*pmrA*/*pmrB*) in the resistant strains, which leads to the hypothesis that PmrAB regulates the colistin susceptibility in A. baumannii. Since then, *pmrA*/*pmrB* mutations have been found in a large number of colistin-resistant A. baumannii isolates, and the mutations were found to constitutively induce the expression of *pmrA*, thereby self-regulating the transcription of *pmrCAB* and transferring phosphoethanolamine (pEtN) to lipid A. These events enable modification of lipid A in the 4′-phosphate group site (16). Chin et al. (17) revealed that the PmrA-PmrB two-component system can simultaneously regulate the transcription of the deacetylase NaxD and the modification of lipid A by deacetylated β-galactosamine, resulting in the loss of colistin susceptibility. The LpxACD genes are responsible for coding enzymes involved in the first three steps of lipid A synthesis. Inactivation of *lpxA* or *lpxC* makes colistin unable to exert antibacterial activity and leads to resistance (18, 19).

In the present work, we obtained a large number of colistin-resistant mutants by screening a transposition mutant library and enriching mutation and by challenging bacterial cultures with increasing concentrations of colistin. Through genome sequencing analysis and genotypic remodeling, we found two novel mutations, *pmrA*^I13M^ and *pmrB*^Q270P^, that conferred colistin resistance, and report here for the first time that the *miaA*^I221V^ mutation was able to enhance the level of *pmrA*^P102R^-mediated colistin resistance.

## RESULTS

### Isolation of colistin-resistant mutants.

**FIG 1:**
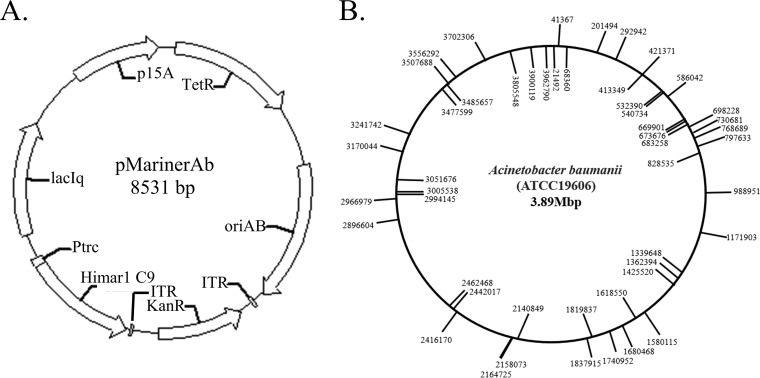
The transposon plasmid map and genetic map of *mariner* transposon insertions. (A) pMarinerAb plasmid map. p15A is the replication origin of E. coli. oriAb is the replicon of A. baumannii from pWH1266. Ptrc is used to control the transposase gene “Himar1 C9” expression derived from Haematobia irritans. The transposase recognizes the terminal inverted repeats (TIRs) to perform the excision of the transposon DNA body, which is inserted into a TA target site. TetR is the tetracycline resistance marker for pMarinerAb plasmid-transferred selection. KanR is a transposon of the kanamycin resistance gene used for isolation of transposon mutants. (B) Transposon insertion sites in A. baumannii mutants. Insertions in the plus orientation are marked on the circle exterior. Insertions in the minus orientation are marked on the circle interior. Numbers indicate the precise point of insertion according to genome sequence data for A. baumannii ATCC 19606.

The constructed pMarinerAb transposon plasmid was electroporated into A. baumannii ATCC 19606, and transposon mutagenesis was performed on LB agar containing IPTG (isopropyl-β-d-thiogalactopyranoside) (1 mM) and kanamycin (50 μg/ml). To verify the random insertion of the transposon, 50 independent transposon insertions were sequenced by reverse PCR (Fig. 1B); among the 50 insertions, 25 were in the plus orientation and the others were in the minus orientation, indicating that the transposon had inserted randomly into the genome of A. baumannii.

A total of 51 colistin-resistant mutants were isolated from the random mutant library that had been spread on LB agar containing colistin (8 μg/ml). Of the 51 mutants, 40 showed colistin MIC between 4 and 64 μg/ml compared to the MIC of 1 μg/ml for the ATCC 19606 parental strain.

In order to obtain a highly resistant mutant strain, the wild-type ATCC 19606 strain was cultured in LB broth and challenged with increasing concentrations of colistin. When the concentration of colistin reached 32 μg/ml, the growth of the cultures was very light, and those cultures were then spread on LB agar containing 64 μg/ml colistin. After incubation for 16 h at 37°C, five highly resistant mutants were isolated that showed colistin MICs ranging from 256 μg/ml to 512 μg/ml. Thus, highly colistin-resistant mutants can be obtained by stepwise challenge with increasing concentrations of colistin.

### Colistin resistance was not associated with the transposon insertion.

In order to analyze the relationship between the transposon insertions and the colistin resistance phenotype, we attempted to complement the resistant phenotype conferred by the colistin-resistant transposon mutants. First, inverse PCR was carried out to identify the genes which had been interrupted in the colistin-resistant mutants. It appeared that double or triple insertions were present in these mutants. Double insertion in Clostridium difficile was also reported previously (20). However, after introduction of the wild-type gene into the mutant in which the same gene has been mutagenized by transposon insertion, the colistin MICs were not restored (Table 1).

**TABLE 1:** Colistin susceptibility in transposon mutants and complemented A. baumannii strains

Strain	Inserted mutation	Complementing gene	Colistin MIC (μg/ml)
ATCC 19606			1–2

Ab-1	Ab19606_02033	None	32–64
		Ab19606_02033	16–32

Ab-3	Ab19606_03164	None	32–64
		Ab19606_03164	32

Ab-4	Ab19606_03163	None	32
		Ab19606_03163	16

Ab-6	Ab19606_02571	None	64
		Ab19606_02571	16–32

Ab-51	Ab19606_00589	None	4–8
		Ab19606_00589	4

Ab-2	Ab19606_01194, Ab19606_03164	None	32
		Ab19606_01194, Ab19606_03164	16

Ab-5	Ab19606_02811, Ab19606_02489	None	32–64
		Ab19606_02811, Ab19606_02489	32

Ab-12	Ab19606_02311, Ab19606_03288	None	32
		Ab19606_02311, Ab19606_03288	16

Ab-15	Ab19606_02311, Ab19606_00589, Ab19606_03288	None	32
		Ab19606_02311, Ab19606_00589, Ab19606_03288	8–16

Ab-30	Ab19606_00589, Ab19606_03740	None	4
		Ab19606_00589, Ab19606_03740	4

Ab-34	Ab19606_00589, Ab19606_03740	None	16–32
		Ab19606_00589, Ab19606_03740	16

Ab-39	Ab19606_00589, Ab19606_00436, Ab19606_00964	None	64
		Ab19606_02311, Ab19606_00589, Ab19606_03288	32

To rule out a polar effect of the transposon insertion, a gene(s) inactivated by transposon insertions was knocked out by homologous recombination from wild-type strain ATCC 19606. With these mutants, the colistin MICs were increased by a maximum of 4 times compared with the level seen with ATCC 19606, far less than MICs of the colistin-resistant transposon mutants (Table 2). Even though the MICs were not as high as those seen with the colistin-resistant transposon mutants, all of them were above the clinical breakpoint of 2 μg/ml, identifying them all as colistin resistant. Therefore, those transposon insertion sites were associated with colistin resistance. Since spontaneous resistant mutants can be selected after A. baumannii is exposed to colistin (16, 21), we suspected that there might have been another mutation(s), besides the transposon insertions, that was responsible for the resistant phenotype.

**TABLE 2:** Colistin susceptibility in transposon mutants and genotypic remodeling A. baumannii strains

Strain	Colistin MIC (μg/ml)
ATCC 19606	1–2
Ab-1	32–64
ATCC 19606 (ΔAb19606_02033)	2
Ab-3	32–64
ATCC 19606 (ΔAb19606_03164)	2–4
Ab-4	32
ATCC 19606 (ΔAb19606_03163)	1–2
Ab-6	64
ATCC 19606 (ΔAb19606_02571)	4
Ab-51	4–8
ATCC 19606 (ΔAb19606_00589)	1–2
Ab-2	32
ATCC 19606 (ΔAb19606_01194, ΔAb19606_03163)	4
Ab-5	32–64
ATCC 19606 (ΔAb19606_02811, ΔAb19606_02489)	2
Ab-12	32
ATCC 19606 (ΔAb19606_02311, ΔAb19606_03288)	4
Ab-15	32
ATCC 19606 (ΔAb19606_02311, ΔAb19606_00589, ΔAb19606_03288)	4
Ab-30	4
ATCC 19606 (ΔAb19606_00589, ΔAb19606_03740)	2–4
Ab-34	16–32
ATCC 19606 (ΔAb19606_00589, ΔAb19606_03740)	4
Ab-39	64
ATCC 19606 (ΔAb19606_02311, ΔAb19606_00589, ΔAb19606_03288)	4

### Whole-genome sequence analysis of the colistin resistance mutants.

To verify whether there are other mutations in the colistin-resistant transposon mutants, complete genome sequencing of 17 colistin-resistant mutants was performed and the resulting sequences were compared with the sequence of the wild-type parent. Point mutations were found in 14 different genes (Table 3), including synonymous mutations in Ab19606_00265 (*mgl*, encoding l-methionine gamma-lyase), Ab19606_1028 (*entC*, isochorismate synthase), Ab19606_3396 (*murI*_2, glutamate racemase), and Ab19606_03754 (replication C family protein). The amino acid substitution in Ab19606_00697 (*pmrA*, transcriptional regulatory protein) or Ab19606_00698 (*pmrB*, sensor protein) appeared in 15 different mutants, with novel amino acid substitutions of *pmrA*^I13M^ and *pmrB*^Q270P^ being reported for the first time. The other eight mutant genes were Ab19606_02965 (encoding a hypothetical protein), Ab19606_01384 (*miaA*, tRNA dimethylallyltransferase), Ab19606_01260 (*betI*_2, HTH-type transcriptional regulator), Ab19606_00066 (*iclR*, transcriptional repressor), Ab19606_01675 (*shlB*_1, hemolysin transporter protein), Ab19606_03709 (*ptk*, tyrosine-protein kinase, Ab19606_00970 (*aroP*_3, aromatic amino acid transport protein), and Ab19606_00971 (*pstS*, phosphate-binding protein). The MiaA gene encodes the tRNA modification enzyme tRNA dimethylallyl diphosphate transferase, and MiaA is a posttranscriptional regulator. MiaA mutants lacking the ms^2^i^6^A-37 and i^6^A-37 tRNA modifications exhibit pleiotropic phenotypes, including mutator and antimutator effects on spontaneous mutation frequencies (22). It is essential for the expression of virulence factors in A. tumefaciens and S. flexneri (23), and it slows the growth rates of Escherichia coli (24) and Yersinia pseudotuberculosis (25) when inactivated. The transcriptional regulators of the IclR family are widely present in Gram-negative bacteria and play an important role in efflux pumps, carbon source utilization, and quorum sensing. It was also reported previously that IclR family transcriptional regulator ABUW_1848 is required for A. baumannii AB5075 virulence (26). Similarly, *ptk* is also associated with virulence and capsular formation in A. baumannii (27), while *shlB* is a virulence-related factor in Serratia marcescens (28). However, no connection between colistin resistance and these eight genes has been reported.

**TABLE 3:** Amino acid changes in colistin-resistant mutants compared to the parental strain

Strain	MIC (μg/ml)	Amino acid change in:									
		*pmrA*	*pmrB*	Ab19606_02965	*miaA*	*betI*_*2*	*iclR*	*shlB*_*1*	*Ptk*	*aroP*_*3*	*pstS*
ATCC 19606	1–2										
Ab256-1	512	P102R		D106E							
Ab256-2	512	P102R		D106E	I221V						
Ab256-3	512	P102R		D106E		L147P					
Ab256-4	512	P102R		D106E							
Ab256-5	512	P102R		D106E							
Ab-1	32–64		Q270P				Y49H	R403H	D569N		
Ab-2	32–64		Q270P				Y49H	R403H	D569N		
Ab-3	32–64		Q270P				Y49H	R403H	D569N		
Ab-4	32		Q270P				Y49H	R403H	D569N		
Ab-5	32–64		Q270P				Y49H	R403H	D569N		
Ab-6	64		T235N				Y49H	R403H	D569N		
Ab-12	32		P233S				Y49H	R403H	D569N		
Ab-15	32		P233S				Y49H	R403H	D569N		
Ab-30	4						Y49H	R403H	D569N		
Ab-34	16–32		P233S				Y49H	R403H	D569N	N137S	Y114C
Ab-39	64	I13M					Y49H	R403H	D569N		
Ab-51	4						Y49H	R403H	D569N		

### Reconstruction site-directed mutant strains exhibit different colistin MICs.

To assign the contribution of point mutation to the resistance of A. baumannii to colistin, we used homologous recombination technology to construct the site-directed mutant strains in wild-type strain ATCC 19606. The results showed that the only point mutation of *pmrA* or *pmrB* caused an 8× or 32× increase in resistance to colistin, respectively (Table 4). PmrA^I13M^ mutation increased the colistin MIC to 32 μg/ml, which was almost the same as the level seen with colistin-resistant transposon mutant Ab-39 containing the *prmA*^I13M^ mutation. The colistin MICs resulting from the *pmrB*^P233S^, *pmrB*^T235N^, and *pmrB*^Q270P^ mutations were also the same as those measured for the corresponding transposon mutants, indicating that *pmrA*/*pmrB* mutations alone can lead to resistance. The MIC seen with the *pmrA*^P102R^ mutation was 32 μg/ml, which was 16-fold lower than those measured for Ab256. In the background of the ATCC 19606 (*pmrA*^P102R^) strain, the mutations of Ab19606_02965^D106E^, *miaA*^I221V^, and *betI*_2^L147P^ were iteratively investigated. The results showed that there was no effect on colistin MICs compared to that seen with the parental ATCC 19606 (*pmrA*^P102R^) strain. When *miaA*^I221V^ was combined with ATCC 19606 (*pmrA*^P102R^), the MIC for colistin increased from 32 μg/ml to 128 μg/ml, whereas with the *miaA*^I221V^ mutation alone, the colistin MIC was 0.5 to 1.0 μg/ml (Table 4). Thus, the *miaA*^I221V^ mutation has a synergistic/additive effect on colistin resistance in the context of *pmrA*^P102R^ mutation.

**TABLE 4:** Colistin susceptibility in reconstruction site-directed mutant strains

Strain	Colistin MIC (μg/ml)
ATCC 19606	1–2
ATCC 19606 (*pmrA*^I13M^)	16–32
ATCC 19606 (*pmrA*^P102R^)	32
ATCC 19606 (*pmrB*^P233S^)	8–16
ATCC 19606 (*pmrB*^T235N^)	16
ATCC 19606 (*pmrB*^Q270P^)	32
ATCC 19606 (Ab19606_02965^D106E^)	1
ATCC 19606 (*miaA*^I221V^)	0.5–1
ATCC 19606 (*betI*_2^L147P^)	0.5–1
ATCC 19606 (*iclR*^Y49H^)	1–2
ATCC 19606 (*shlB*^R403H^)	2
ATCC 19606 (*ptk*^D569N^)	2–4
ATCC 19606 (*aroP*^N137S^)	4–8
ATCC 19606 (*pstS*^Y114C^)	2
ATCC 19606 (*pmrA*^P102R^, Ab19606_02965^D106E^)	32
ATCC 19606 (*pmrA*^P102R^, *miaA*^I221V^)	128
ATCC 19606 (*pmrA*^P102R^, *betI*_2^L147P^)	32

The *aroP*^N137S^ mutation, which encoded an aromatic amino acid transport protein, resulted in the reconstitution of resistance, and the MIC of colistin was 4 to 8 μg/ml. The mutation was located in the amino acid permease domain. It was reported previously that deletion of *aroP* increased the accumulation of extracellular aromatic amino acids (29). It is possible that expression of the amino acid biosynthesis genes led to production of osmolytes that mediated osmotic tolerance through membrane and protein stabilization (30).

### Analysis of *pmrCAB* transcription in different strains.

**FIG 2:**
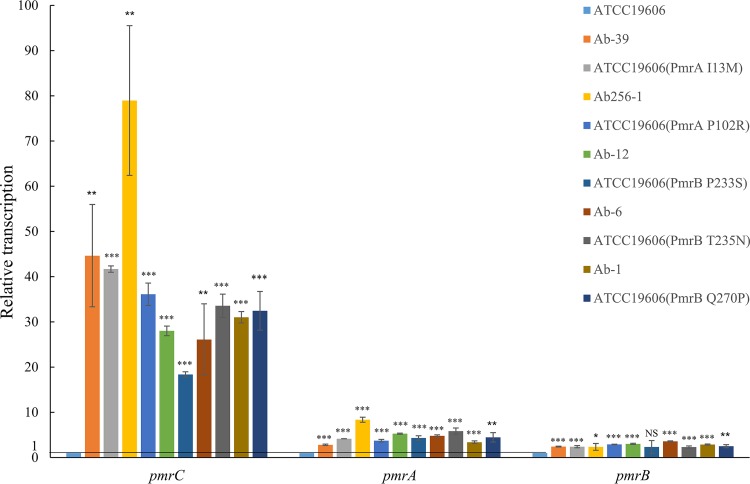
Quantification of relative transcriptional levels of *pmrCAB* genes in colistin-resistant A. baumannii mutants versus the ATCC 19606 parental strain. The experiments were performed in triplicate. Bars represent means ± standard deviations. *, 0.01≤*P ≤ *0.05; **, *P < *0.01; ***, *P < *0.001; NS, not significant.

According to the phenotypic results corresponding to the reconstituted resistance, the *pmrAB* mutation is the main cause of drug resistance. A colistin-resistant phenotype would be associated with increased expression of the *pmrC* gene, which encodes the protein that adds phosphoethanolamine to lipid A. We studied the transcription levels of the *pmrCAB* operon in different strains, including Ab-39, Ab256-1, Ab-12, Ab-6, and Ab-1 and the reconstructed, site-directed mutant strains ATCC 19606 (*pmrA*^I13M^), ATCC 19606 (*pmrA*^P102R^), ATCC 19606 (*pmrB*^P233S^), ATCC 19606 (*pmrB*^T235N^), and ATCC 19606 (*pmrB*^Q270P^), in comparison to its level in ATCC 19606. The results showed that the levels of transcription of *pmrCAB* in the mutant strains were higher than in the parental ATCC 19606 strain, with the transcription of *pmrC* of Ab256-1 being 78.96-fold higher than the level seen with ATCC 19606 (Fig. 2). These data indicate that higher transcription level of *pmrC* could be responsible for elevating the MIC of colistin.

### Lipid A was modified in colistin-resistant strains.

**FIG 3:**
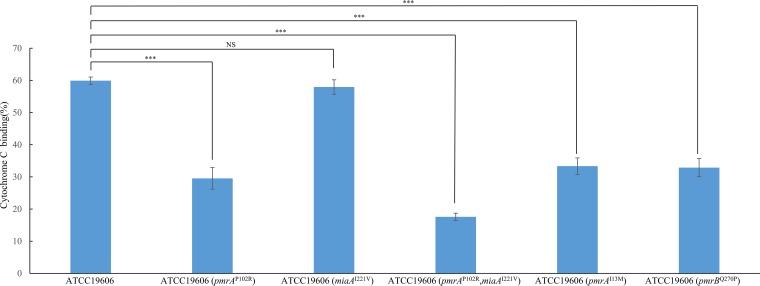
Analysis of cytochrome *c* binding to different A. baumannii strains, including ATCC 19606, ATCC 19606 (*pmrA*^P102R^), ATCC 19606 (*miaA*^I221V^), ATCC 19606 (*pmrA*^P102R^, *miaA*^I221V^), ATCC 19606 (*pmrA*^I13M^), and ATCC 19606 (*pmrB*^Q270P^). The experiments were performed in triplicate. Bars represent means ± standard deviations. *, 0.01≤*P ≤ *0.05; **, *P < *0.01; ***, *P < *0.001; NS, not significant.

To investigate whether lipid A is modified in colistin-resistant strains, we measured the cytochrome *c* binding ability of the sensitive ATCC 19606 strain and the resistant mutants. Cytochrome *c* is a highly cationic protein that binds to anionic surfaces in a charge-dependent manner and exhibits a characteristic level of absorbance. The results showed that mutations in *pmrA*^P102R^, *pmrA*^P102R^ plus *miaA*^I221V^, *pmrA*^I13M^, or *pmrB*^Q270P^ decreased cationic cytochrome *c* binding to A. baumannii, indicating a lower negative charge of the colistin-resistant strain than of the parental ATCC 19606 strain (Fig. 3).

**FIG 4:**
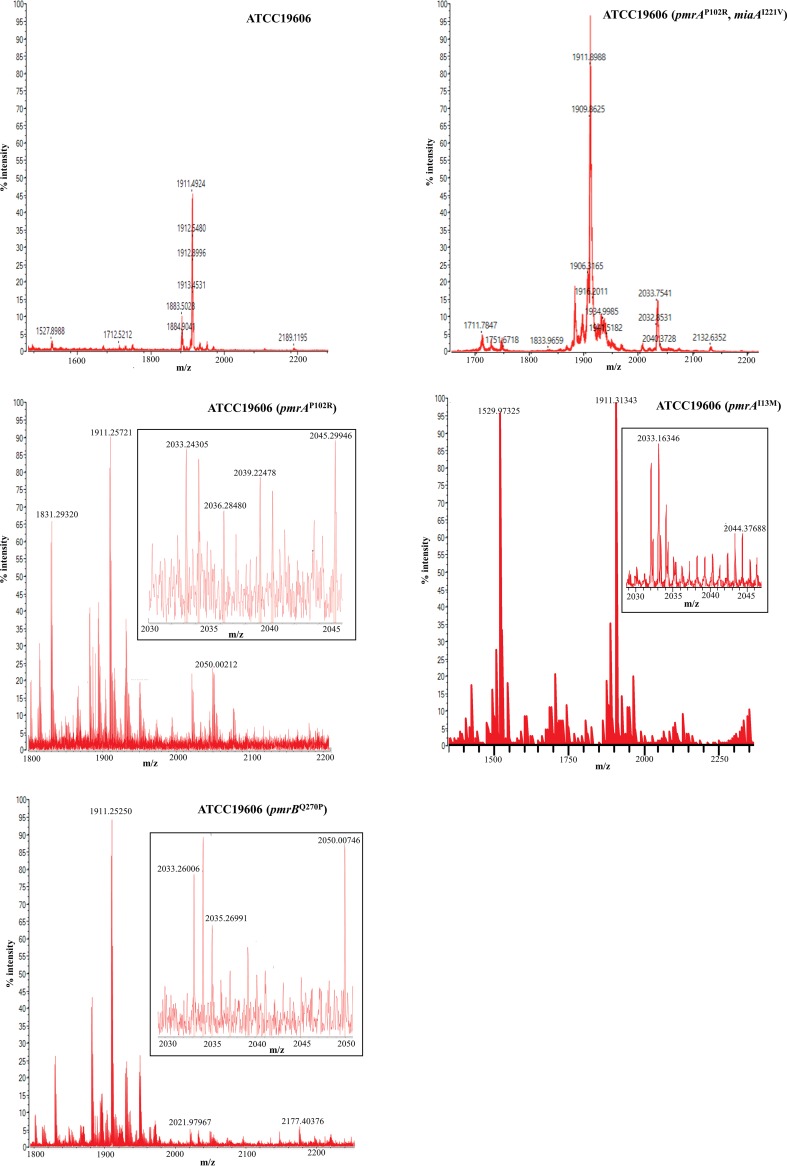
Negative-ion mode MALDI-TOF MS mass spectra of lipid A from colistin-susceptible and colistin-resistant A. baumannii strains ATCC 19606, ATCC 19606 (*pmrA*^P102R^, *miaA*^I221V^), ATCC 19606 (*pmrA*^P102R^), ATCC 19606 (*pmrA*^I13M^), and ATCC 19606 (*pmrB*^Q270P^). The insets show partial enlarged views to display the ions at *m*/*z* 2,033.

Lipid A of the resistant strains and of the sensitive ATCC 19606 strain was analyzed by mass spectrometry (MS). Analysis of lipid A isolated from ATCC 19606 (Fig. 4) showed that an abundant [M-H]- ion was at *m*/*z* 1,910 and was identified as representing a singly deprotonated lipid A structure that contains two phosphate groups and seven acyl chains (i.e., diphosphoryl hepta-acylated lipid A) (31). Of note, ions at *m*/*z* 2,033 were absent from this spectra. In contrast, resistant strains ATCC 19606 (*pmrA*^P102R^, *miaA*^I221V^), ATCC 19606 (*pmrA*^P102R^), ATCC 19606 (*pmrA*^I13M^), and ATCC 19606 (*pmrB*^Q270P^) (Fig. 4) displayed not only [M-H]- at *m*/*z* 1,910 but also ions at *m*/*z* 2,033, which indicates a phosphoethanolamine (pEtN) modification of lipid A.

## DISCUSSION

As the last resort for the treatment of multidrug-resistant A. baumannii infection, the mechanism of action of colistin and control of resistance are important for preserving and improving efficacy. At present, the number of research samples is very limited and the samples were collected mainly from clinical practice where exact controls are difficult to obtain. In order to fully understand the mechanism of A. baumannii resistance to colistin, we obtained a large number of colistin-resistant mutants from a random transposon mutant library and from successive challenges using gradually increased concentrations of colistin. Although the transposon mutant technique is widely used in bacterial genetics studies, our results indicated that using a transposon mutant library to screen mutants resistant to colistin is not a good choice, because A. baumannii spontaneous chromosomal mutations are readily selected and result in resistant mutants after exposure to colistin.

The *pmrA*^I13M^, *pmrA*^P102A^, *pmrB*^P233S^, *pmrB*^T235N^, and *pmrB*^Q270P^ mutations found in this study all conferred resistance to colistin, resulting in 8-to-32-times-higher MICs than wild-type strain ATCC 19606. Two mutations, *pmrA*^I13M^ and *pmrB*^Q270P^, were never reported previously for colistin resistance. The colistin resistance-related mutations in *pmrA* were located in the sulfatase domain, and the mutations in *pmrB* were in the histidine kinase domain. Mutations in the PmrA-PmrB two-component system are not only the main cause of colistin resistance in A. baumannii but are also found in Pseudomonas aeruginosa, E. coli, and *Salmonella* (16). Mutations in *pmrA-mrB* facilitate phosphorylation of the PmrB receptor kinase, which in turn activates *pmrA*. The activated PmrA regulates the expression of the *pmrC* gene that encodes the phosphoethanolamine transferase. In the present work, the transcription level of *pmrC* in colistin-resistant strains was shown to be higher than that in the ATCC 19606 wild-type strain via real-time quantitative PCR (RT-qPCR), and the lipid A modification was detected by matrix-assisted laser desorption ionization–time of flight mass spectrometry (MALDI-TOF MS) analysis in drug-resistant strains.

In addition, our work showed that the *miaA* mutation synergistically/additively enhanced the ability of *pmrA*^P102R^ to resist colistin, although the increase in the MIC of colistin conferred by the *miaA*^I221V^ mutation itself was minimal. MiaA acts as a posttranscriptional regulator that has been previously reported to affect cell growth and virulence. For example, Marceau et al. (25) reported that the deletion of *miaA* in Y. pseudotuberculosis caused a downregulation of the *phoP* transcriptional level and that the PhoP-PhoQ two-component system in Y. pseudotuberculosis is essential for lipid A modification. PhoP-PhoQ upregulation could increase the synthesis of 4-aminoarabinose to modify lipid A, which results in colistin resistance. As the PhoP-PhoQ two-component system is absent in A. baumannii, we speculate that another unknown gene(s) regulated by *miaA* might affect colistin resistance. Identification of these genes and elucidation of their role in colistin resistance warrant further systematic studies as follow-up investigations.

In summary, we found that two novel mutations, *pmrA*^I13M^ and *pmrB*^Q270P^, are involved in A. baumannii colistin resistance and that a *miaA* mutation further enhanced *pmrA*^P102R^-mediated resistance to colistin. These findings will help expand our knowledge of the mechanism of colistin resistance in A. baumannii.

## MATERIALS AND METHODS

### Bacterial strains and culture conditions.

The strains used in this study are listed in Table 5. Escherichia coli DH5α was used as the cloning host for plasmid construction. Luria-Bertani (LB) medium was used to propagate A. baumannii and E. coli. The LB plates contained 1.5 g/liter agar. Kanamycin and tetracycline were purchased from Amresco, and colistin E was purchased from Apeloa. Colistin E MICs were measured by the broth microdilution method according to the Clinical and Laboratory Standards Institute (CLSI) guidelines (32).

**TABLE 5:** Strains and plasmids used in this study

Strain(s) or plasmid	Characteristics^*a*^	Source or reference
Strain(s)		
E. coli DH5α	General cloning host strain	Vazyme
A. baumannii strain 19606	Wild type	ATCC
A. baumannii Ab-1, Ab-2, Ab-3, Ab-4, Ab-5, Ab-6, Ab-12, Ab-15, Ab-30, Ab-39, Ab-51	Colistin-resistant mutants derived from transposon mutant library of ATCC 19606	This study
A. baumannii Ab256-1, Ab256-2, Ab256-3, Ab256-4, Ab256-5	Colistin-resistant mutants derived from successive challenges of ATCC 19606 with increased concentrations of colistin	This study

Plasmid		
pWH1266	Acinetobacter-E. coli shuttle vector, Amp^r^, Tet^r^	37
pSU2718	p15A, Cat^r^	38
pSUTetAB	Acinetobacter-E. coli shuttle vector, Tet^r^	This study
pTrc99a	pMB1, *aadA*, *lacIq*	39
pSUTetABtrc	*trc* promoter cloned into pSUtetAB, Tet^r^	This study
pMTL-SC1	ColE1 ORI, *ermB*, pBP1 ORI, *traJ*, PtcdB, Himar1C9, *catP*	20
pPIC9k	Pichia pastoris expression plasmid, Amp^r^, Kan^r^	Invitrogen
pMarinerAb	Himar1C9- and Kan-resistant fragment cloned into pSUTetABtrc, Tet^r^	This study
pAT03	pMMB67EH with FLP recombinase, Amp^r^	33
pAT04	pMMB67EH with RecAb system, Tet^r^	33
pMD19-T simple	General cloning vector, Amp^r^	Takara
pKD4	bla FRT-kan-FRT	40
pMDK	FRT-kan-FRT cloned into pMD19-T simple	This study
pIJ773	Aac(3)IV	41
pAT03-Apr	pMMB67EH with FLP recombinase, Apra^r^	This study

a Amp^r^, ampicillin resistance; Apra^r^, apramycin resistance; Cat^r^, chloramphenicol resistance; Kan^r^, kanamycin resistance; Tet^r^, tetracycline resistance.

### Screening of colistin-resistant mutants *in vitro*.

The transposon mutant library was spread on an LB plate containing colistin (8 μg/ml) and was cultured at 37°C. Colonies were picked for MIC determinations, and a MIC value of ≥4 μg/ml was used to score colistin-resistant mutants (Ab-) for study (Table 5).

The exponential phase of a wild-type A. baumannii ATCC 19606 culture was treated with 4 μg/ml colistin for 24 h. Then, the concentration of colistin was successively increased to 32 μg/ml (e.g., 8, 16, and 32 μg/ml stepwise in the following round of challenge). The culture was then spread on LB agar containing colistin (64 μg/ml) and grown at 37°C. Colonies were picked for MIC determination, and a MIC value of ≥256 μg/ml was used as the criterion for highly resistant mutants (Ab256-x) for further study (Table 5).

### pMarinerAb plasmid construction.

Using the pSU2718 vector as a template and 2718-F/2718-R as primers, the P2718 fragment containing the p15A replicon fragment was amplified. pWH1266 vector was used as a template and Tet-F/Tet-R as primers for PCR amplification of the Tet fragment containing a tetracycline resistance gene. The P2718 and the Tet fragments were ligated using the Gibson assembly method to generate the pSUTet plasmid. With pWH1266 vector used as the template and AB-F/AB-R as primers, the AB-Rep fragment containing Acinetobacter baumannii plasmid replication origin was amplified by PCR. The AB-Rep fragment was then cloned into the pSUTet plasmid PvuI site by the Gibson assembly method to generate the pSUTetAB plasmid. The pTrc99a plasmid was used as a template and trc-F/trc-R as primers for PCR amplification of the Ptrc fragment containing the Ptrc promoter and lacIq. The Ptrc fragment was cloned into the SmaI site of pSUTetAB using the Gibson assembly method to generate the pSUTetABtrc plasmid.

The Himar1, Kan, ITR, and SUTETABtrc fragments were PCR amplified, using Himar-F/Himar-R, Kan-F/Kan-R, ITR-F/ITR-R, and pSU-F/pSU-R as primers and pMTL-SC1, pPIC9k, pMTL-SC1, and pSUTetABtrc plasmids as the templates, respectively. These four fragments were ligated via the Gibson assembly method to generate the pMarinerAb plasmid (Fig. 1A).

### Complementary plasmid construction.

The complement gene fragments containing the natural promoter and the open reading frame of the gene of interest were amplified by PCR using the ATCC 19606 genome as the template and the -CF/-CR series as primers (see Table S1 in the supplemental material). The fragments were then cloned into the SmaI site of pSUTetAB plasmid via the Gibson assembly method to generate pSUTetAB series plasmids (Table S2).

10.1128/mSphere.00895-19.1 TABLE S1 Oligonucleotides used in this study. Download Table S1, DOCX file, 0.02 MB. Copyright © 2020 Sun et al. 2020 Sun et al. This content is distributed under the terms of the Creative Commons Attribution 4.0 International license.

10.1128/mSphere.00895-19.2 TABLE S2 Plasmids used in this study. Download Table S2, DOCX file, 0.02 MB. Copyright © 2020 Sun et al. 2020 Sun et al. This content is distributed under the terms of the Creative Commons Attribution 4.0 International license.

### Construction of resistance marker curing plasmids.

The pAT03 plasmid contains a recombinase for eliminating a resistance gene on the chromosome. As the ATCC 19606 strain used in this study was not sensitive to ampicillin, the ampicillin resistance gene of pAT03 needed to be replaced with the apramycin (Apr) resistance gene. The apramycin resistance gene was derived from the pIJ773 plasmid and PCR amplified as an Apr fragment using Apr-F/Apr-R primers. The Apr fragment was cloned into the PvuI site of pAT03 to generate pAT03-Apr plasmid.

### Transposon mutant library construction.

The *mariner* pMarinerAb plasmid was transferred into the wild-type A. baumannii ATCC 19606 strain by electroporation and was grown on LB agar containing tetracycline (15 μg/ml). ATCC 19606/pMarinerAb was placed into sterile phosphate-buffered saline (PBS), spread on an LB plate containing kanamycin (50 μg/ml) and IPTG (1 mM), and cultured at 30°C to induce expression of the Himar1 C9 transposase in pMarinerAb to generate the transposon mutant library.

### Gene knockouts and introduction of amino acid changes by homologous recombination.

The construction was performed as described previously (33). Briefly, the template plasmid was constructed by combining fragments containing a resistance marker and two 500-bp regions of homologous DNA sequences that flank upstream and downstream of the targeted gene. A kanamycin resistance marker was PCR amplified from pKD4 using Kan-F0/Kan-R0 primers and cloned into pMD19-T simple to create pMDK. Using the ATCC 19606 genome as the template and the -UP-KF/-UP-KR, -DN-KF/-DN-KR series as primers, the upstream and the downstream homologous fragments, both about 500 bp, were PCR amplified. Then, the upstream and downstream homologous fragments were cloned into the KpnI/SalI and SmaI/NotI sites of pMDK, respectively, to generate the gene knockout template plasmid pMDK::(Gene) series (Table S2). The ATCC 19606 genome was used as the template, and the -UP-MF1/-UP-MR1, -UP-MF2/-UP-MR2 series were used as primers for PCR amplification of the -UP1M and -UP2M fragments. The -UP1M and -UP2M fragments were then used as the templates and teh -UP-MF1/-UP-MR2 series as primers for overlap PCR amplification of the upstream homologous fragments containing the amino acid mutation(s). Similarly, the downstream homologous fragments were PCR amplified using the ATCC 19606 genome as the template and the -DN-MF/-DN-MR series as primers. The upstream and downstream homologous fragments were also cloned into the KpnI/SalI and SmaI/NotI sites of pMDK, respectively, to generate the site-directed mutagenesis template plasmids pMDK-(gene) series (Table S2).

Primers M13F/M13R binding outside the regions of homology were used to PCR amplify the genes of interest and for insertion of a kanamycin cassette. The PCR products were digested with DpnI, purified by gel extraction, and concentrated to 5 μg (5 μl) using a speed vacuum concentrator (Xiangyi, Changsha, Hunan, China) and then electroporated into 100 μl competent cells containing pAT04 in a 2-mm-path-length cuvette at 2.5 kV. The cells were transferred immediately to 900 μl of prewarmed LB medium, allowed to recover at 37°C with 220 rpm shaking for 1 h, plated on LB agar containing kanamycin and tetracycline, and incubated for 18 h.

For curing of the kanamycin cassette, pAT03-Apr plasmid was electroporated into the positive-testing mutant strains obtained as described above, after which expression of the FLP recombinase was induced with 1 mM IPTG at 37°C on LB agar. Loss of kanamycin marker was observed during differential plating of colonies on agar containing or lacking kanamycin and was further verified by PCR and DNA sequencing.

### Preparation of competent cells and electroporation.

A. baumannii containing pAT04 plasmid was streaked on LB agar containing tetracycline (15 μg/ml) and cultured at 37°C, and a single colony was picked and inoculated into LB liquid medium containing tetracycline and cultured overnight at 37°C with 220 rpm shaking. One milliliter of the preculture was inoculated into 50 ml of LB medium containing IPTG (1 mM) and tetracycline (10 μg/ml) in a shaking flask. The cultures were harvested and made electrocompetent when the optical density at 600 nm (OD_600_) reached approximately 0.8 to 1.0. The cells were chilled on ice for 30 min and collected by centrifugation at 2,667 × *g* at 4°C for 10 min, and then they were washed three times with 25 ml of cold 10% glycerol. Competent cells were resuspended in 500 μl of 10% glycerol, and 100-μl aliquots were dispensed into 1.5-ml sterile centrifuge tubes. The competent cells should be freshly prepared before use.

Before electroporation, 100 μl of competent cells was mixed with 5 μg of DNA on ice and then transferred into precooled 2-mm-path-length electroporation cuvettes. Electroporation was performed at 2.5 kV for 5 ms. The cells were transferred immediately to 900 μl of LB liquid medium and were recovered at 37°C with 220 rpm shaking for 1 h. The recovered cells were then spread on LB agar with the corresponding antibiotic at 37°C until colonies appeared.

### Comparative genome sequencing of A. baumannii ATCC 19606 and the colistin-resistant mutants.

Bacterial strains were grown at 37°C in LB broth until the OD_600_ reached ∼1.0. Cells were harvested by centrifugation for 1 min at 12,000 × *g*, and genomic DNA was isolated using a bacterial genomic DNA miniprep kit (Axygen, Hangzhou, Zhejiang, China) following the manufacturer’s protocol. The complete genome sequencing procedure was performed using an Illumina HiSeq 2000 system by the Shanghai Haoyu Biotechnology Company. Multiple alignments were performed for the wild-type strain and the derived mutants.

### RT-qPCR.

A 4-ml volume of the overnight culture was harvested via centrifugation at 13,523 × *g* for 1 min. Total RNA was extracted with an RNA extraction kit according to the instructions of the manufacturer (CoWin Biosciences). RNA (1 μg) was reverse transcribed into cDNA with random primers with a ReverTra-Plus kit from Toyobo (Shanghai, China). The product was quantified via real-time PCR using a CFX96 thermal cycler (Bio-Rad). The reaction mixture (20 μl) contained Power SYBR green PCR master mix (Bio-Rad) and 0.4 μM gene-specific -F(RT)/-R(RT) primer series as shown in Table S1. The PCR parameters were one cycle of 95°C for 2 min followed by 40 cycles of 95°C for 20 s, 55°C for 20 s, and 72°C for 15 s. The *rpoB* housekeeping gene was used as a reference to normalize the relative amounts of mRNA, and ATCC 19606 was used to normalize the transcriptional level of each strain (34).

### Determination of cell surface charge by cytochrome *c* binding assay.

To evaluate the bacterial surface charge, we used cytochrome *c* (purchased from Sigma), a highly cationic eukaryotic protein that binds to anionic surfaces in a charge-dependent manner and exhibits a characteristic level of absorbance. A. baumannii strains were grown in LB liquid medium to an OD_600_ of ∼1.0, determinations were performed as previously described (35), and the incubation time was adjusted to 35 min.

### Lipid A isolation and purification.

The hot phenol-water method (36) was used and slightly modified to extract lipopolysaccharide (LPS). In short, bacteria were grown at 37°C in LB broth until the OD_600_ reached approximately 0.8 to 1.0. Bacterial cells were harvested by centrifugation (2,667 × *g*, 10 min) and were washed three times with 0.9% NaCl. After the final step of centrifugation with washing, cells were resuspended with 4 volumes of sterile water. The bacterial suspension was subjected to freeze-and-thaw in a −70°C refrigerator 5 times. After the final thawing step, bacterial cells were broken by the use of ultrasonic homogenizer (Scientz JY92-IIN; Ningbo, Zhejiang, China) for 2 s with 3 s intervals, for a total of 20 min, at 325 W power. Thereafter, an equal volume of 90% phenol was added to the cell lysate. The mixture was incubated in a 68°C water bath for 20 min and then centrifuged at 2,667 × *g* for 10 min to collect the upper aqueous phase. Then, 10 ml of sterile water was added to the lower phase followed by incubation in a 68°C water bath for 20 min and centrifugation as described above. The upper aqueous phase was collected and combined with the first-step aqueous phase and then dialyzed in a dialysis bag for 3 to 4 days against distilled water that was changed every 5 h. After dialysis, the solution was concentrated to 5 ml with polyethylene glycol 20000 (Sinopharm).

Purification of lipopolysaccharide and extraction of lipid A were performed as previously reported (31) with appropriate modifications. The LPS solution was treated with DNase (Yuanye) and RNaseA (Yuanye) at 50 μg/ml and incubated at 37°C for 4 h in a water bath. Proteinase K (Sangon Biotech) was added to reach a final concentration of 50 μg/ml, and the reaction mixture was incubated at 56°C for 1 h in a water bath. Then, the solution was boiled in water for 10 min, cooled to room temperature, and centrifuged at 2,667 × *g* for 30 min. Six volumes of absolute ethanol was added to the supernatant, and the reaction mixture was incubated at 4°C overnight and then centrifuged at 7,254 × *g* for 20 min. After the alcohol had evaporated, the pellet was dissolved in sterile water and then frozen and freeze-dried. The sample was then washed with chloroform/methanol (2:1) 3 times. After chloroform/methanol evaporation, purified LPS was converted to lipid A by mild-acid hydrolysis with 1% sodium dodecyl sulfate (SDS) at pH 4.5 followed by boiling in water for 1 h. After being cooled, the sample was dried at 60°C overnight, washed three times with 100 μl of sterile water and 500 μl of acidified ethanol, and centrifuged at 2,000 × *g* for 10 min. The sample was then washed three times with 500 μl of 95% ethanol and centrifuged at 2,000 × *g* for 10 min. Finally, the insoluble lipid A content was extracted in 200 μl of a mixture of CHCl_3_/CH_3_OH/H_2_O (3:1:0.25) and then the supernatant was purged with nitrogen to obtain a solid sample of lipid A.

### Analysis of lipid A by mass spectrometry.

The MS testing was performed in the Shanghai Jiao Tong University Analysis and Testing Center. For matrix-assisted laser desorption ionization–time of flight (MALDI-TOF) MS analysis, a MALDI-7090 instrument (Kratos Analytical Ltd., Shimadzu Corporation, Manchester, England) was equipped with a solid-state ultrafast UV laser (Nd: YAG 355-nm wavelength). A total of 200 laser shots were used for each measurement position. External calibration was performed using peaks from a matrix mix (DHB [2 5-dihydroxybenzoic acid], CHCA [α-cyano-4-hydroxycinnamic acid], and TOF mix). The parameters were set as follows: laser intensity, 40 to 60 per unit area [u.a.]; repetition rate laser frequency, 200 Hz; mass range, 500 to 5,000 Da; 250 shots accumulated per profile; laser beam diameter set at 100 μm. MALDI-MS data were viewed and processed using MALDI solution software. A 0.5-μl volume of sample was spotted, and then 0.5 μl of 20 mg/ml matrix 9-aminoacridine–ethanol was spotted.
