# Supplementary material for: New Mutations Involved in Colistin Resistance in Acinetobacter baumannii
Source: mSphere. 2020 Apr 1;5(2):e00895-19. doi: 10.1128/mSphere.00895-19 (PMC7113586; doi:10.1128/mSphere.00895-19)
Supplement: TABLE S1 [file mSphere.00895-19-st001.docx]

| Primer name | Sequence (5’→3’) |
| --- | --- |
| 2718-F | CATGATCGTGCTCCTGTCGTTCTTCCTTAGCTCCTGAAAATCTC |
| 2718-R | GAAGACGAAAGGGCCTCGTGATCGGGGCGTAATTTTTTTAAGGC |
| TET-F | GCCTTAAAAAAATTACGCCCCGATCACGAGGCCCTTTCGTCTTC |
| TET-R | GAGATTTTCAGGAGCTAAGGAAGAACGACAGGAGCACGATCATG |
| AB-F | ATTCGCCATTCAGGCTGCGCAACTGTTGGGAAGGGCGGATCGGATTTTAACATTTTGCG |
| AB-R | TTTCGCCAGCTGGCGTAATAGCGAAGAGGCCCGCACCGAACAGCTCGCCAAGATCGTAG |
| trc-F | AGCTATGACCATGATTACGAATTCGAGCTCGGTACCCGGTGCACTCTCAGTACAATCTG |
| trc-R | GCTTGCATGCCTGCAGGTCGACTCTAGAGGATCCCCCCGAGCTCGAATTCCATGGTCTG |
| Himar-F | GTGAGCGGATAACAATTTCACACAGGAAACAGACATGGAAAAAAAGGAATTTCGTG |
| Himar-R | GACACAACGTGGCTTTCCCCCCCTCTCAAATTCAAGTTTATCGC |
| Kan-F | GCGATAAACTTGAATTTGAGAGGGGGGGAAAGCCACGTTGTGTC |
| Kan-R | CGGCAAATGTGAAATCCGTCACCAACCAATTAACCAATTCTGATTAG |
| ITR-F | CTAATCAGAATTGGTTAATTGGTTGGTGACGGATTTCACATTTGCCG |
| ITR-R | AGCTTGCATGCCTGCAGGTCGACTCTAGAGGATCCCCGTGACGTCGACTCTAGAGGATC |
| pSU-F | GTCTGTTTCCTGTGTGAAATTG |
| pSU-R | GGGGATCCTCTAGAGTCGACCTGC |
| Kan-INV-F | GATGATGCATGGTTACTCACCACTG |
| Kan-INV-R | ATTCCGTCAGCCAGTTTAGTCTGAC |
| 2033-CF | AGCTATGACCATGATTACGAATTCGAGCTCGGTACCCGATGCGGGTGGGGCTTTTAAGAG |
| 2033-CR | GCTTGCATGCCTGCAGGTCGACTCTAGAGGATCCCCCATATTAAGCACCCTATGAACTTC |
| 3164-CF | AGCTATGACCATGATTACGAATTCGAGCTCGGTACCCATCAATCATTCCAAGCCCCTG |
| 3164-CR | GCTTGCATGCCTGCAGGTCGACTCTAGAGGATCCCCGAATCGACTCAAATTGCTCTGC |
| 3163-CF | AGCTATGACCATGATTACGAATTCGAGCTCGGTACCCCAATAAGCCTGAAAATATGCGTTC |
| 3163-CR | GCTTGCATGCCTGCAGGTCGACTCTAGAGGATCCCCACGAATCTGAAGATGTACCAGAAG |
| 2571-CF | AGCTATGACCATGATTACGAATTCGAGCTCGGTACCCGTGTTTATTTTGGCTATTTTCCTG |
| 2571-CR | GCTTGCATGCCTGCAGGTCGACTCTAGAGGATCCCCACGTGCAATGTCTTATATTCCAGC |
| 589-CF | AGCTATGACCATGATTACGAATTCGAGCTCGGTACCCGGTTTCTAACACCGATTTACT |
| 589-CR | GCTTGCATGCCTGCAGGTCGACTCTAGAGGATCCCCACAGCAGTGCTGTCGAAATCATC |
| 1194-CF | GTGATCAGGGGCTTGGAATGATTGATGGGTACCGGCTGCGATTCTACGCTATCATAAC |
| 1194-CR | ACAGGAAACAGCTATGACCATGATTACGAATTCGCTCTTTTGGAGCTGAATGATCTGG |
| 2811-CF | AGCTATGACCATGATTACGAATTCGAGCTCGGTACCCACTTACCGCATTTTTAACTTGGTC |
| 2811-CR | GTCTTAGTGTGTCTTGGAGTGGCCAACCTTGTTTAGGGTCTATTCC |
| 2489-CF | GGAATAGACCCTAAACAAGGTTGGCCACTCCAAGACACACTAAGAC |
| 2489-CR | GCTTGCATGCCTGCAGGTCGACTCTAGAGGATCCCCGCTGTGGATAAACAAATAGAAGTC |
| 3289-CF | AGCTATGACCATGATTACGAATTCGAGCTCGGTACCCCTTTTGCACAACGTGTAATCATTG |
| 3289-CR | CAAAGCATGCTGTTCATAGAGGAGCGGAGTGACTAACCCCACTGAGTC |
| 2311-CF | GACTCAGTGGGGTTAGTCACTCCGCTCCTCTATGAACAGCATGCTTTG |
| 2311-CR | GCTTGCATGCCTGCAGGTCGACTCTAGAGGATCCCCAGATCAGGGCTGGGTGAACGGTG |
| 3740-CF | TTATGAGTAAATCGGTGTTAGAAACCGGGTACCGCAACGCTGAAAATTTACCCTCCTC |
| 3740-CR | ACAGGAAACAGCTATGACCATGATTACGAATTCGGCTTGTTTACAATCGAGTGGGAG |
| 964-CF | TTATGAGTAAATCGGTGTTAGAAACCGGGTACCGAGTTGGGAAAATTTAGAGAAGTAG |
| 964-CR | GTAGAACACCATCATCAAATTTTGGAGCGCGATGATACCTAATTTTGC |
| 436-CF | GCAAAATTAGGTATCATCGCGCTCCAAAATTTGATGATGGTGTTCTAC |
| 436-CR | ACAGGAAACAGCTATGACCATGATTACGAATTCGATTTCTTCAGACATTTATTTCCCC |
| Apr-F | GTGATAACACTGCGGCCAACTTACTTCTGACAACGGAATAGGAACTTATGAGCTCAGCC |
| Apr-R | GTGCAAAAAAGCGGTTAGCTCCTTCGGTCCTCCGCTGACGCCGTTGGATACACCAAGG |
| KAN-F0 | CCGGTACCGCGTCGACGTGTAGGCTGGAGCTGCTTC |
| KAN-R0 | CCGCGGCCGCGCCCCGGGATGGGAATTAGCCATGGTCC |
| M13F | GTTGTAAAACGACGGCCAG |
| M13R | CAGGAAACAGCTATGAC |
| 2033-UP-KF | CCGGTACCGATTACTTAGCAGATTTTCCTTATG |
| 2033-UP-KR | GCGTCGACGTGAGACCTGAATCACTGAAAATG |
| 2033-DN-KF | GCCCCGGGCGAGCAAGTAGAATTAATATGGC |
| 2033-DN-KR | CCGCGGCCGCCGGTACTCTTTGCATTGAGCAAC |
| 3164-UP-KF | CCGGTACCGTTAAATGGGATGCTGGTTCAAG |
| 3164-UP-KR | GCGTCGACGACATATAAATGCCATTTTCAGAG |
| 3164-DN-KF | GCCCCGGGACAAGTATCTGATAAGCAAAATGC |
| 3164-DN-KR | CCGCGGCCGCATTGCCAAAAGGGGTAGATGTGAC |
| 3163-UP-KF | CCGGTACCATTATGGATGATCAAGATGGCCTG |
| 3163-UP-KR | GCGTCGACCAATTGCAATAATTGTTGGGTCG |
| 3163-DN-KF | GCCCCGGGGTCGGTATTCTAAATCTTGAAACTC |
| 3163-DN-KR | CCGCGGCCGCCTGAAAATATGCGTTCCATTTAAAC |
| 2571-UP-KF | CCGGTACCAAGTGAGCATTGCTCTCATAGTCG |
| 2571-UP-KR | GCGTCGACATGGATCTTTTTTATAGTCTCGGTC |
| 2571-DN-KF | GCCCCGGGACTACCGTTTACGTGCTTACCATG |
| 2571-DN-KR | CCGCGGCCGCCCAAATCAAACTCTAGCGAATC |
| 589-UP-KF | CCGGTACCGAATAAAAACTAACTTGAAAAGTG |
| 589-UP-KR | GCGTCGACAATGGTGACTCATATAAAGTGAAAG |
| 589-DN-KF | GCCCCGGGATCGAAATTTTTGCGAGTGCATAC |
| 589-DN-KR | CCGCGGCCGCGTAACGCACGTAAGGTATCGGTC |
| 1194-UP-KF | CCGGTACCGAAGAACTATAGCGGCGGTGGTC |
| 1194-UP-KR | GCGTCGACCAAACCATGAGTCACGTGTATCG |
| 1194-DN-KF | GCCCCGGGACACACGAATCATTTCAAGTGGTAG |
| 1194-DN-KR | CCGCGGCCGCCACGTCTTGCAAGAGACTCAGCG |
| 2811-UP-KF | CCGGTACCGTAGCTATTTTCGAATGCTTTGC |
| 2811-UP-KR | GCGTCGACAGTATTCGGGTCGAAACACAAATG |
| 2811-DN-KF | GCCCCGGGCGAGCCACAAATTATTTTTGCCG |
| 2811-DN-KR | CCGCGGCCGCGAGTATGTGCCATTGAACTAAAC |
| 2489-UP-KF | CCGGTACCGGGCACCTACCCTCATATGTCAC |
| 2489-UP-KR | GCGTCGACGATTCTTCAATAGCCATCAAAAC |
| 2489-DN-KF | GCCCCGGGCAATAATTAGGGTTTACCCTTTTAG |
| 2489-DN-KR | CCGCGGCCGCGTGCAATAACGATATCGCCGTGC |
| 2311-UP-KF | CCGGTACCGCCAGCCCAATACCGTCTGAAC |
| 2311-UP-KR | GCGTCGACCCATAAACATGTTACGGGCTTTG |
| 2311-DN-KF | GCCCCGGGCATCTGGAATTAACATTAAAGGTG |
| 2311-DN-KR | CCGCGGCCGCGCTACCTGTAGTTTTGATGGTTC |
| 3289-UP-KF | CCGGTACCCTGAAGAATATGAAGGTGGTGATC |
| 3289-UP-KR | GCGTCGACATCACATCTAAACAATGATTACACG |
| 3289-DN-KF | GCCCCGGGCTCAAATTGACTTCGATTTTACTC |
| 3289-DN-KR | CCGCGGCCGCCAGAAATAATGACGCCATCACGAC |
| 3740-UP-KF | CCGGTACCAAGGACGCGGAACATGCCTCATG |
| 3740-UP-KR | GCGTCGACCTTGACCAGGTGCGTGGCAATG |
| 3740-DN-KF | GCCCCGGGATCCGAGAAGCGGCTGTCGTTAAC |
| 3740-DN-KR | CCGCGGCCGCGCTCGTCAGTCAAACAGACCCAG |
| 436-UP-KF | CCGGTACCGAAACGGTACGTAAATTCTATGAG |
| 436-UP-KR | GCGTCGACAGATCCTTTATCGCTATTCCCCG |
| 436-DN-KF | GCCCCGGGATCCAATTGCACGCGCTTATATC |
| 436-DN-KR | CCGCGGCCGCGATTATTGGCTATGTAAATGCTTC |
| 964-UP-KF | CCGGTACCCGCGATGATACCTAATTTTGCAAG |
| 964-UP-KR | GCGTCGACGCATTTCATCGTTTGAAACTTG |
| 964-DN-KF | GCCCCGGGAATAAAAGTAATTTTCCGGGTCC |
| 964-DN-KR | CCGCGGCCGCACAGCGTAAAGGTAATCCCACTTG |
| pmr-DN-MF | CGCCCGGGTTACGTAAGCTCTTGTTTCACTTG |
| pmr-DN-MR | CCGCGGCCGCACAGGTTGTGAATGGATACACTC |
| pmr-UP-MF1 | CCGGTACCCATGGATCAACTTTATCTATACGC |
| pmrA13-UP-MR1 | CGTTGATTCTGCCATCATAAAATCATC |
| pmrA13-UP-MF2 | GATGATTTTATGATGGCAGAATCAACG |
| pmrA13-UP-MR2 | GCGTCGACTCACGCTCTTGTTTCATTTAAATG |
| pmrA102-UP-MR1 | CATCAAACTCATAACGTTTAATTAA |
| pmrA102-UP-MF2 | TTAATTAAACGTTATGAGTTTGATG |
| pmrB233-UP-MR1 | GTGCAGTCACAAGTGTTCGTAATTC |
| pmrB233-UP-MF2 | GAATTACGAACACTTGTGACTGCAC |
| pmrB235-UP-MR1 | AAGTTCAGTGCATTCACAGGTGTTC |
| pmrB235-UP-MF2 | GAACACCTGTGAATGCACTGAACTT |
| pmrB270-UP-MR1 | ATGCTAAAAGCGGAGTCACCAAATG |
| pmrB270-UP-MF2 | CATTTGGTGACTCCGCTTTTAGCAT |
| 2965-UP-MF1 | CCGGTACCCCAGTTGAAAGAAGGCGTTGAAATG |
| 2965-UP-MR1 | CTAAAGTTTCAACTTCATGACCAAAT |
| 2965-UP-MF2 | ATTTGGTCATGAAGTTGAAACTTTAG |
| 2965-UP-MR2 | GCGTCGACCAAGTATCTTTTAAAAGTGAGTCAT |
| 2965-DN-MF | CGCCCGGGGTTAATTTTGCCCTAGTATGAGG |
| 2965-DN-MR | CCGCGGCCGCCGGCCATACATGAACTTAAGCATG |
| miaA-UP-MF1 | CCGGTACCATTACTCCACTTGAGGTCTATTCG |
| miaA-UP-MR1 | CAAAAAACCGACATCCCACATTTTG |
| miaA-UP-MF2 | CAAAATGTGGGATGTCGGTTTTTTG |
| miaA-UP-MR2 | GCGTCGACGTGTGCAAATTTTGCTTTATCCATA |
| miaA-DN-MF | CGCCCGGGGCACACTGTCTTTTTATAATTTTT |
| miaA-DN-MR | CCGCGGCCGCAAGAAACAGAGATGGACCGCAGG |
| betI-UP-MF1 | CCGGTACCACAGGCTGGATAATGATCAAAATTG |
| betI-UP-MR1 | GATAAGTTCGGGATTATACTTTTG |
| betI-UP-MF2 | CAAAAGTATAATCCCGAACTTATC |
| betI-UP-MR2 | GCGTCGACAGAACTGGCTCCTATGTTTTAAAAT |
| betI-DN-MF | CGCCCGGGCCTATGTTTTTAATATATGGTGGTG |
| betI-DN-MR | CCGCGGCCGCGAAATTGCTAAATTAGCTGCTAGAC |
| iclR-DN-MF | CCGGTACCAGACCATGTACCTTCAACGAAAGC |
| iclR-DN-MR | GCGTCGACGCGTAAATTGATAGAGGTTTAGTAG |
| iclR-UP-MF1 | CGCCCGGGACGCTATAAATGTAAGATGAAGGTG |
| iclR-UP-MR1 | GCTCACCAAGTGGCGGTGTACTTTG |
| iclR-UP-MF2 | CAAAGTACACCGCCACTTGGTGAGC |
| iclR-UP-MR2 | CCGCGGCCGCGTTTTTTTATGGCTATTGATTAGC |
| shlB-DN-MF | CCGGTACCAGGTTATTCATACTGCCATACGTCT |
| shlB-DN-MR | GCGTCGACAATTTGGTAAAGAAACTTCGTCTTC |
| shlB-UP-MF1 | CGCCCGGGGTTCATAAGTTAGCAAAATCATTCC |
| shlB-UP-MR1 | CCCCATCCCATGACGGTAATCGATG |
| shlB-UP-MF2 | CATCGATTACCGTCATGGGATGGGG |
| shlB-UP-MR2 | CCGCGGCCGCACGTTTCCTACTGTTTTTGTCGTAG |
| ptk-UP-MF1 | CCGGTACCGCAGAGCTTGAGCAGAAACAAGCTG |
| ptk-UP-MR1 | GCATATCGGCATTGATCAATAGTAC |
| ptk-UP-MF2 | GTACTATTGATCAATGCCGATATGC |
| aroP-DN-MF | CCGGTACCACAGCTAAATATATTGCTCAACCG |
| aroP-DN-MR | GCGTCGACGTCACTTCCTGTACACCTTTATTC |
| aroP-UP-MF1 | CGCCCGGGCCAACATTGGAGATAATGTTATG |
| aroP-UP-MR1 | CAAGTTAATGCCGCTAATTAAAAT |
| aroP-UP-MF2 | ATTTTAATTAGCGGCATTAACTTG |
| aroP-UP-MR2 | CCGCGGCCGCCCATTTTGCCCCAGTATTTGTTCGC |
| pstS-DN-MF | CCGGTACCAGTCGTATGTTGCATGGTTTAGCAG |
| pstS-DN-MR | GCGTCGACGGGAAATATGACAAACGTTTAAAAT |
| pstS-UP-MF1 | CGCCCGGGCCCATTAAATCTAAATAGCAAAGG |
| pstS-UP-MR1 | ACAATACCGTCACAACCAATTTTG |
| pstS-UP-MF2 | CAAAATTGGTTGTGACGGTATTGT |
| pstS-UP-MR2 | CCGCGGCCGCCAGCTAAAACTTTAGCGCGTTCTGC |
| rpoB-F (RT) | ATGCCGCCTGAAAAAGTAAC |
| rpoB-R (RT) | TCCGCACGTAAAGTAGGAAC |
| pmrA-F (RT) | GGTGTTGCTGCTCTTTGACG |
| pmrA-R (RT) | GGTGGAATGGGTCAATAACG |
| naxD-F (RT) | ACCGATCAATCCCCCTCATT |
| naxD-R (RT) | GTTCGCCATAATTGGTCAGT |
| pmrC-F (RT) | CTCTTTACGCTTTGTTTTATGGAC |
| pmrC-R (RT) | GTAAAAAGTAAAACACCGACCA |
