# Supplementary material for: New Mutations Involved in Colistin Resistance in Acinetobacter baumannii
Source: mSphere. 2020 Apr 1;5(2):e00895-19. doi: 10.1128/mSphere.00895-19 (PMC7113586; doi:10.1128/mSphere.00895-19)
Supplement: TABLE S2 [file mSphere.00895-19-st002.docx]

| Plasmid | Characteristics | Source |
| --- | --- | --- |
| pMDK::2033 | Donor plasmid for Ab19606_02033 deletion | this study |
| pMDK::3164 | Donor plasmid for Ab19606_03164 deletion | this study |
| pMDK::3163 | Donor plasmid for Ab19606_03163 deletion | this study |
| pMDK::2571 | Donor plasmid for Ab19606_02571 deletion | this study |
| pMDK::589 | Donor plasmid for Ab19606_00589 deletion | this study |
| pMDK::1194 | Donor plasmid for Ab19606_01194 deletion | this study |
| pMDK::2811 | Donor plasmid for Ab19606_02811 deletion | this study |
| pMDK::2489 | Donor plasmid for Ab19606_02489 deletion | this study |
| pMDK::2311 | Donor plasmid for Ab19606_02311 deletion | this study |
| pMDK:: 3289 | Donor plasmid for Ab19606_03289 deletion | this study |
| pMDK::3740 | Donor plasmid for Ab19606_03740 deletion | this study |
| pMDK::436 | Donor plasmid for Ab19606_00436 deletion | this study |
| pMDK::964 | Donor plasmid for Ab19606_00964 deletion | this study |
| pSUTetAB | *Acinetobacter*-*E*. *coli* shuttle vector, Tet^r^ | this study |
| pSUTetAB-2033 | pSUTetAB carrying Ab19606_02033 with native promoter | this study |
| pSUTetAB-3164 | pSUTetAB carrying Ab19606_03164 with native promoter | this study |
| pSUTetAB-3163 | pSUTetAB carrying Ab19606_03163 with native promoter | this study |
| pSUTetAB-2571 | pSUTetAB carrying Ab19606_02571 with native promoter | this study |
| pSUTetAB-589 | pSUTetAB carrying Ab19606_00589 with native promoter | this study |
| pSUTetAB-1194-3164 | pSUTetAB carrying Ab19606_01194 and Ab19606_03164 with native promoters | this study |
| pSUTetAB-2489-2811 | pSUTetAB carrying Ab19606_02489 and Ab19606_02811 with native promoters | this study |
| pSUTetAB-2311-3289 | pSUTetAB carrying Ab19606_02311 and Ab19606_03289 with native promoters | this study |
| pSUTetAB-589-2311-3289 | pSUTetAB carrying Ab19606_00589, Ab19606_02311 and Ab19606_03289 with native promoters | this study |
| pSUTetAB-589-3740 | pSUTetAB carrying Ab19606_00589 and Ab19606_03740 with native promoters | this study |
| pSUTetAB-436-589-964 | pSUTetAB carrying Ab19606_00436, Ab19606_00589 and Ab19606_00964 with native promoters | this study |
| pMDK-pmrA^I13M^ | Donor plasmid for site-directed mutagenesis of *pmrA*^I13M^ | this study |
| pMDK-pmrA^P102R^ | Donor plasmid for site-directed mutagenesis of *pmrA*^P102R^ | this study |
| pMDK-pmrB^P233S^ | Donor plasmid for site-directed mutagenesis of *pmrB*^P233S^ | this study |
| pMDK-pmrB^T235N^ | Donor plasmid for site-directed mutagenesis of *pmrB*^T235N^ | this study |
| pMDK-pmrB^Q270P^ | Donor plasmid for site-directed mutagenesis of *pmrB*^Q270P^ | this study |
| pMDK-2965^D106E^ | Donor plasmid for site-directed mutagenesis of Ab19606_02965^D106E^ | this study |
| pMDK-miaA^I221V^ | Donor plasmid for site-directed mutagenesis of *miaA*^I221V^ | this study |
| pMDK-betI_2^L147P^ | Donor plasmid for site-directed mutagenesis of *betI*_2^L147P^ | this study |
| pMDK-iclR^Y49H^ | Donor plasmid for site-directed mutagenesis of *iclR*^Y49H^ | this study |
| pMDK-shlB^R403H^ | Donor plasmid for site-directed mutagenesis of *shlB*^R403H^ | this study |
| pMDK-ptk^D569N^ | Donor plasmid for site-directed mutagenesis of *ptk*^D569N^ | this study |
| pMDK-aroP^N137S^ | Donor plasmid for site-directed mutagenesis of *aroP*^N137S^ | this study |
| pMDK-pstS^Y114C^ | Donor plasmid for site-directed mutagenesis of *pstS*^Y114C^ | this study |
